# Bibliographical Notices

**Published:** 1844-01-13

**Authors:** 


### BIBLIOGRAPHICAL NOTICES.

*Lectures on the Principles and Practice of Physic, delivered at King's College, London.* By THOMAS WATSON, M. D., Fellow of the Royal College of Physicians, Physician to the Middlesex Hospital, and formerly Fellow of St. John's College, Cambridge. 8vo. pp. 92. Philadelphia: 1844.

Many of these Lectures were published in the *London Medical Gazette*, and were so well received, that the author issued a complete series of them during the last year, from which the greater part of the present volume has been reprinted. Some of them had already been laid before the profession in this country in the pages of different periodicals, and had acquired for the author no little celebrity as a practised observer and an accomplished teacher. We know not, indeed, of any work of the same size that contains a greater amount of interesting and useful matter. The author is evidently well acquainted with everything that appertains to the principles and practice of medicine, and has incorporated the stores of his well-stocked mind, in the work before us, so ably and so agreeably, that it is impossible for the interest of the reader to flag for a moment.

"They" [the lectures] "do not profess," says the author, "to present a formal and complete treatise on the Practice of Physic, much less to exhaust the various subjects upon which they touch. His chief hope is that they may prove useful as a text book for students."

That they are well adapted for such a purpose, all must admit; but their sphere of usefulness may extend much beyond this. We are satisfied, indeed, that no physician, well-read and observant as he may be, can rise from their perusal without having added largely to his stock of valuable information.

Where so many topics are discussed, it is not easy to now which to select; we shall therefore take one or two at random. On the subject of Malaria, the author adopts the true, we think, but by no means general, opinion—that it is not the product of vegetable decomposition.

"Where there is much heat and much moisture," he observes, "there we usually find also much and rank vegetation, and much vegetable dissolution and decay. The belief was as natural, therefore, as it has been general, that the putrefaction of vegetable matters was somehow or other requisite to the formation of the poison that exists so commonly in swampy situations. This belief has descended, almost unquestioned, from the time of Lancisi, and it obtains almost universal acceptance, I fancy, among physicians of the present day. Yet very strong facts have been adduced to show, that the decomposition of vegetable substances is only an accidental, though a frequent, *accompaniment* of the miasm, and not by any means an essential condition of its evolution. In the first place, the decomposition of vegetable matter goes on abundantly without the production of malaria. The rotting cabbage-leaves of Covent Garden, and those which taint the air of the streets from the neglected dust-holes of London, during the hot weather of summer, give rise to no ague. The same may be said of the putrefying and offensive seaweed, which is deposited in large quantities upon some very healthy parts of our sea-coast."

After adducing a number of facts to prove the converse that vegetation is not necessary, he concludes:

"Now these facts, and facts like these seem to prove that the malaria and the product of vegetable decomposition are two distinct things. They are often in company with each other, but they have no necessary connection."

These views are certainly corroborative of the communications which appeared in this Journal a short time ago, under the title of "Contributions to the History of Malaria."

On the contested subject of continued fever, as regards the separate and distinct diseases of typhoid and typhus, Dr. Watson is evidently of opinion that they are but different forms of adynamic fever; and he concludes, "upon the whole, that although an inflammatory state of the solitary and aggregate glands, which strew the surface of the mucous membrane of the alimentary canal, is not the essence of fever, yet that it is a very frequent companion of continued fever."

---

*Principles of Forensic Medicine.* By WILLIAM A. GUY, M. D., Professor of Forensic Medicine, King's College, London, Physician to King's College Hospital, &c. &c. Parts 1 and 2, pp. 391, 12mo. London, 1843.

This will be an excellent compendium of medical jurisprudence, judging from the two parts already published. The plan, adopted by the author in treating the several subjects, has been, as he states to begin with a short account of the existing provisions and requirements of the law, as relates to Great Britain, avoiding all unnecessary discussion as to the state of the law in former times, and in different countries. This is followed by a brief statement of the main medical questions arising out of the law, which are then investigated under distinct heads; practical rules for medico-legal examinations are appended, and the subjects are illustrated throughout by cases.

The parts already published contain the following subjects: Medical Evidence—Personal Identity—Age—Sex—Impotence—Rape—Pregnancy—Delivery—Fœticide—Infanticide—Legitimacy—Life Assurance—Feigned Diseases—Unsoundness of Mind—Persons Found Dead—Real and apparent Death—Sudden Death—Survivorship—Wounds—Death from Asphyxia—Drowning—Hanging—Strangulation—Suffocation—Death by the inhalation of poisonous gases—Lightning—Cold—Starvation.

The third part which will conclude the work, will treat of Toxicolgy. It will be published on the first of March, 1844.

